# Quantification of Analgesic and Anti-Inflammatory Lipid Mediators in Long-Term Cryopreserved and Freeze-Dried Preserved Human Amniotic Membrane

**DOI:** 10.3390/bioengineering10060740

**Published:** 2023-06-20

**Authors:** Vladimir Vrkoslav, Ingrida Smeringaiova, Natalia Smorodinova, Alzbeta Svobodova, Stepan Strnad, Catherine Joan Jackson, Jan Burkert, Katerina Jirsova

**Affiliations:** 1 The Institute of Organic Chemistry and Biochemistry of the Czech Academy of Sciences, 160 00 Prague, Czech Republic; vrkoslav@uochb.cas.cz (V.V.); stepan.strnad@uochb.cas.cz (S.S.); 2 Laboratory of the Biology and Pathology of the Eye, Institute of Biology and Medical Genetics, First Faculty of Medicine, Charles University and General University Hospital in Prague, 128 01 Prague, Czech Republic; 3 Institute of Histology and Embryology, First Faculty of Medicine, Charles University and General University Hospital in Prague, 128 01 Prague, Czech Republic; 4 2nd Department of Surgery—Department of Cardiovascular Surgery, First Faculty of Medicine, Charles University and General University Hospital in Prague, 128 08 Prague, Czech Republic; alzbeta.svo@seznam.cz; 5 Department of Medical Biochemistry, Oslo University Hospital and Institute of Oral Biology, University of Oslo, 0316 Oslo, Norway; 6 Department of Transplantation and Tissue Bank, University Hospital in Motol, 150 06 Prague, Czech Republic

**Keywords:** amniotic membrane allografts, cryopreserved amniotic membrane, lyophilization, freeze-dried amniotic membrane, N-acylethanolamines, palmitoylethanolamide, tissue banking, mass spectrometry

## Abstract

The aim of this study was to compare concentrations of endogenous N-acylethanolamine (NAE) lipid mediators—palmitoylethanolamide (PEA), oleoylethanolamide (OEA), and anandamide (AEA)—in fresh, decontaminated, cryopreserved, and freeze-dried amniotic membrane (AM) allografts, thereby determining whether AM’s analgesic and anti-inflammatory efficiency related to NAEs persists during storage. The concentrations of NAEs were measured using ultra-high-performance liquid chromatography–tandem mass spectrometry. Indirect fluorescent immunohistochemistry was used to detect the PEA PPAR-α receptor. The concentrations of PEA, OEA, and AEA were significantly higher after decontamination. A significant decrease was found in cryopreserved AM compared to decontaminated tissue for PEA but not for OEA and AEA. However, significantly higher values for all NAEs were detected in cryopreserved samples compared to fresh tissue before decontamination. The freeze-dried AM had similar values to decontaminated AM with no statistically significant difference. The nuclear staining of the PPAR-α receptor was clearly visible in all specimens. The stability of NAEs in AM after cryopreservation was demonstrated under tissue bank storage conditions. However, a significant decrease, but still higher concentration of PEA compared to fresh not decontaminated tissue, was found in cryopreserved, but not freeze-dried, AM. Results indicate that NAEs persist during storage in levels sufficient for the analgesic and anti-inflammatory effects. This means that cryopreserved AM allografts released for transplant purposes before the expected expiration (usually 3–5 years) will still show a strong analgesic effect. The same situation was confirmed for AM lyophilized after one year of storage. This work thus contributed to the clarification of the analgesic effect of NAEs in AM allografts.

## 1. Introduction

The human amniotic membrane (AM) has long been used to treat wounds in ophthalmology, dermatology, and surgery [1]. The therapeutic effect of AM allografts originates in the high content of growth factors, cytokines, extracellular matrix components, and the presence of pluripotent stem cells [2,3]. AM is typically obtained from the placenta after caesarean section, prepared by manual separation from the underlying chorionic membrane, cleaned, decontaminated, and stored. For transplantation purposes, serological tests for potentially transmissible diseases (human immunodeficiency virus, hepatitis B and C viruses, and syphilis) must be negative both at the time of tissue collection and when repeated after 180 days [4,5].

Based on the number of papers published on the use of placental membranes for grafting in the PubMed database, the most common storage method for AM is cryopreservation [6,7,8]. Reports over the last few years have suggested that cryopreservation has been slowly supplanted by freeze-dry methods (lyophilized, vacuum freeze-dried), which are particularly common in wound healing treatment [9,10,11]. However, for ophthalmosurgery, both storage methods for AM are used equally [10,12,13].

The method of AM processing may affect its therapeutic properties, i.e., induction of granularization and re-epithelialization [14], reduction of fibrosis [15], pain [16,17], inflammation [18], antimicrobial [19], and antiviral [20] properties, and also pro-angiogenic [21,22] and anti-angiogenic [23,24] features. 

Cryopreservation preserves the biological activity and structure of tissue. Before the cryopreservation storage, AM is chemically decontaminated and usually placed in a mixture of culture medium and glycerol [25,26].

Freeze-dried AM is prepared in a lyophilizer, where the tissue is gently dried using a high-powered vacuum to a final water content of 5–10% [27]. The crucial advantage of freeze-drying compared to cryopreservation is that AM allografts can be stored and transported at room temperature. Disadvantages include tissue destruction and a decrease in protein levels and activity if gamma-sterilization is used for terminal sterilization [28,29,30,31]. Recently, radiation has been substituted for a less destructive chemical decontamination technique under clean room conditions. Thus, the AM structure is preserved with no serious structure and composition deterioration [11,32]. 

During clinical studies, apart from accelerating healing, the most outstanding effect of AM application is rapid wound pain alleviation [17,33,34]. The exact mechanism of analgesic action of AM was not known for a long time and no directly acting component was characterized [35]. Pain relief was thought to be due to the good adhesion of the AM graft to the wound surface, which covers free nerve endings, maintains wound moisture, and releases anti-inflammatory substances that can indirectly relieve pain [35,36,37,38,39]. 

Recently, we have detected and analyzed the levels of *N*-acylethanolamides (NAEs), particularly palmitoylethanolamide (PEA), oleoylethanolamide (OEA), and anandamide (AEA), in various placental tissues, mainly in amniotic and chorionic membranes [40]. We suggested that these NAEs are responsible for pain relief and also have an anti-inflammatory effect. These NAEs were represented in AM in the concentration order of PEA > OEA > AEA, and the concentration of all NAEs increased significantly after 24 h decontamination with antibiotic solution [40].

NAEs are widely spread endogenous bioactive lipid mediators derived from complex membrane lipids in response to environmental stimuli, and they play an important role in numerous physiological processes, such as immune function, metabolic regulation, pain, and inflammation [41]. It has been proposed that PEA, the most abundant NAE in vertebrates [41], accumulates in tissues after injury and exerts anti-inflammatory, neuroprotective, analgesic, and anti-nociceptive effects, mainly through the PPAR-α receptor [42,43,44]. All these PEA properties have been demonstrated in human clinical trials [45,46,47], including trials on treatment of chronic pain [46,48]. 

Beside PEA, AEA has also been implicated in possessing anti-nociceptive and anti-inflammatory effects [41,49,50,51]. Similarly, a role for AEA in wound healing also been suggested [41,50]. OEA has mostly anorexigenic properties [52] and its ability to reduce nociceptive responses and inflammation has been shown [45]. 

The aim of this study was to monitor the dependence of changes in the concentration of PEA, AEA, and OEA in AM on the method of tissue storage (cryopreservation and lyophilization) and the duration of storage, in order to find out whether and for how long individual NAEs persist in the tissue prepared for transplantation purposes.

## 2. Materials and Methods

### 2.1. Placenta Retrieval, Decontamination, and Amniotic Membrane Preparation

The study followed the ethics committee’s standards of participating institutions, First Medical Faculty of Charles University, the General Teaching Hospital (GTH), and University Hospital Motol (UHM), all in Prague, and adhered to the tenets set out in the Declaration of Helsinki. Human placentas obtained at elective caesarean section from normal pregnancies were obtained with informed consent after delivery at UHM. Only healthy donors, screened for hepatitis B and C, syphilis, HIV, and C-reactive protein (<10 mg/L), were selected. 

### 2.2. Sample Preparation

#### 2.2.1. Fresh and Decontaminated Samples

**Figure 1:**
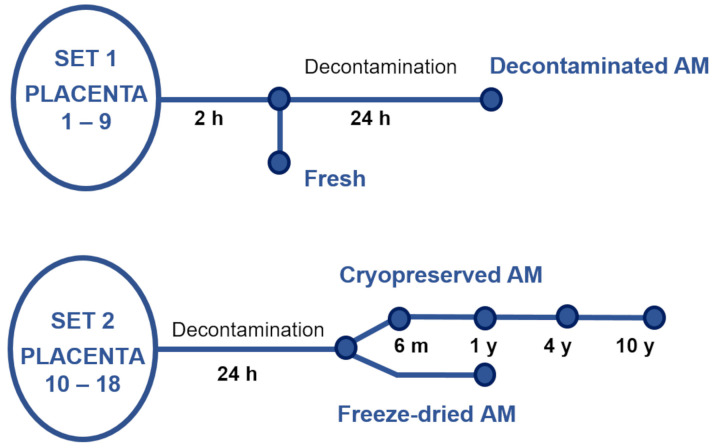
The schema of amniotic membrane (AM) sample preparation. Fresh and decontaminated AM samples were prepared from placenta 1–9 (set 1), and cryopreserved and freeze-dried AM were from placenta 10–18 (set 2).

Nine placentas (P1–P9, set 1), Figure 1, were used for preparation of fresh and decontaminated samples. Within two hours after the delivery, AM samples were processed and stored using procedures and protocols valid for the preparation of AM for transplantation purposes [53]. Shortly after, AM was prepared by manual dissection in the biohazard cabinet. The tissue was washed using sterile saline (0.9% *w*/*v*, Fresenius Kabi, Langenhagen, Germany) and separated from blood clots. Then, part of the AM was stretched on a mesh support (Sanatyl; Tylex, Letovice, Czech Republic) and cut to 2 × 2 cm. These fresh specimens (with no decontamination) were immediately used for NAE analysis. The other part of the AM (within two hours after the delivery) was placed in decontamination solution BASE 128 (Alchimia s.r.l., Ponte San Nicolò, Padova, Italy) for 24 h at room temperature. Then, the 2 × 2 cm samples were stretched on a mesh support and immediately used for NAE analysis. 

#### 2.2.2. Cryopreserved and Lyophilized Samples

The cryopreserved and freeze-dried samples were obtained from nine other placentas (P10–P18, set 2), Figure 1. Immediately after the delivery, each placenta was placed in decontamination solution BASE 128 for 24 h at room temperature, then the samples were processed as described above, again based on the preparation of AM for transplantation purposes [53]. The samples were cryopreserved or freeze-dried (lyophilized).

All specimens for cryopreservation were immersed in 40 mL of 1:1 mixture of Dulbecco’s Modified Eagle’s Medium (c. n. 32430-027, Gibco Life-Technologies, Invitrogen, Waltham, MA, USA)/glycerol (Glycerolum 85%, Dr. Kulich Pharma s.r.o., Hradec Kralove, Czech Republic), and stored in sterile containers (S245-J, Medfor Products Ltd., Aldershot, UK) at −80 °C until the date of NAE analyses [26,54].

Lyophilized samples were prepared according to a previously published method [55]. Briefly, AM samples on a mesh carrier (washed with physiological saline) were placed in a sterile Petri dish and freeze-dried (lyophilizer VirTis AdVantage Pro™ Laboratory Benchtop Freeze Dryer, Biotrade Instruments, s. r. o., Prague, Czech Republic). The samples were placed on the pre-frozen lyophilizer shelves (set point: −40 °C) and freezing (thermal treatment) was performed for 30 min after cooling to −20 °C. Then, the sample was dried in six steps: (1) shelf: −20 °C, hold: 360 min; (2) shelf: 0 °C, hold: 360 min; (3) shelf: 20 °C, hold: 360 min; (4–5) shelf: 30 °C, hold: 999 min; (6) shelf: 30 °C, hold: 762 min. All the six steps had the same ramp (10 min) and vacuum (200 mTorr) values. After lyophilization, the samples were placed in secondary packaging and stored at room temperature. 

AM cryopreserved specimens were stored for 6 ± 1 month (mean 6.2), 12 ± 2 months (mean 11.9 months), 48 ± 6 months (mean 52 months), and 10 years (±1 year, mean 10.4); freeze-dried samples were stored for six months (±1 months, mean 6.1) and for one year (±2 months, mean 1.1). 

### 2.3. Sample Preparation and UHPLC/MS of NAEs

All specimens (size 2 × 2 cm) were used in triplicates. The experiments were performed in duplicates. Fresh and decontaminated AM samples were washed in saline three times for five minutes and removed from mesh support. The same procedure was applied to freeze dried samples, and to cryopreserved samples after thawing.

Samples were processed according to a previously published protocol [40]. Briefly, samples were mechanically homogenized in cold acetonitrile (HiPerSolv CHROMANORM, LC-MS grade, Leuven, Belgium) (cut with scissors, 2 min). Then, internal standard solution PEA-d_4_ (with ≥99% deuterium incorporation, Cayman Chemicals, Ann Arbor, MI, USA) was added (10 µL, 1 µg/mL) to all homogenates. Samples were shaken (4 °C and 800 rpm) and then centrifuged (20 min., 15,000× *g*). Supernatants (extracts) were evaporated to dryness in a vacuum centrifuge set at 0 °C and re-dissolved in 1 mL of 30% (*v*/*v*) methanol (HiPerSolv CHROMANORM, LC-MS grade, Leuven, Belgium) and purified via solid phase extraction following a previously published protocol [40]. Pellets were dried in an evacuated centrifuge (Refrigerated CentriVAp Concentrator, Labconco Corporation, Kansas City, MO, USA) and weighed. Samples were stored at −80 °C until UHPLC/MS.

The UHPLC/MS system consisted of an ExionLC UHPLC AD chromatography system and a QTRAP 6500+ mass spectrometer (both Sciex, Foster City, CA, USA) with an electrospray. A recently published UHPLC/MS method was used [42]. Internal standards PEA-d4 (10 µL at 1 µg/mL) were used to construct seven-point combined NAE calibration curves (OEA (≥98%) and AEA (MaxSpec standard quality) Cayman Chemicals, Ann Arbor, MI, USA; PEA (≥98%), Merck, Darmstadt, Germany), constructed for the relative signal intensity of the analyte (related to area of the internal standard). Peak integration, calibration curve construction, and determination of analyte concentration were performed using Analyst 1.6.3 (Sciex, Darmstadt, Germany). For each AM sample, the concentration of specific NAEs (PEA, OEA, and AEA) was related to the weight of the extracted material.

### 2.4. Immunohistochemistry for PPAR α Receptor

Fluorescent indirect immunohistochemistry [56,57] was used to detect PPAR-α receptor, in fresh, decontaminated, cryopreserved, and freeze-dried AM samples from four placentas. Cryospecimens of human arm skin were used as a positive control [58]. Specimens were washed three times for five minutes in sterile saline and fixed on a mesh support. 

A circle with a diameter of ~1 cm was cut from the 2 × 2 cm samples and placed on a plastic holder, in which incubation took place. Samples were fixed using 4% paraformaldehyde, and then cell membranes were permeabilized using 0.33% Triton X-100 (Sigma-Aldrich, St. Louis, MI, USA) diluted in PBS. Primary antibody (mouse monoclonal antibody PPAR-α, H-2 clone, Santa Cruz Biotechnology sc-398394, diluted 1:500 in 0.1% bovine serum albumin) was applied overnight at room temperature, then samples were washed and secondary antibody Alexa Fluor 488 goat anti-mouse IgG (A11029, Invitrogen, Frederick, MD, USA) was applied overnight at 4 °C and washed three times in PBS. The mesh holder was removed and specimens placed on a slide and mounted with VectaShield-PI (Vector Laboratories, Burlingame, CA, USA). The sample of AM was also used as a negative control (primary antibody omitted). The immunostaining was assessed using a fluorescence microscope (Nikon ECLIPSE Ni-U, Nikon, Tokyo, Japan) at ×200 and ×400 magnifications. The images were obtained with a VDS CD-1300QF (VDS Vosskühler GmbH, Osnabrück, Germany) camera, and for positivity evaluation, image management software (NIS Elements; Laboratory Imaging, Prague, Czech Republic) was used. The percentage of positive cells was calculated in at least 2000 cells.

### 2.5. Statistical Analysis

Each sample was analyzed in triplicate. The resulting average + SD was calculated from 6–9 mean values (depending on the sample) from each triplicate. Mann–Whitney test was used to compare specific NAE concentrations between individual AM samples: fresh (control), decontaminated (control for all stored samples), and cryopreserved or freeze-dried samples, see Table S1 in Supplementary Materials. *p*-values less than 0.05 were considered statistically significant. Statistical analysis was performed using GraphPad Prism 8.0 software (La Jolla, CA, USA).

## 3. Results

### 3.1. NAE Concentrations in Fresh, Cryopreserved and Freeze-Dried Amniotic Membrane

**Figure 2:**
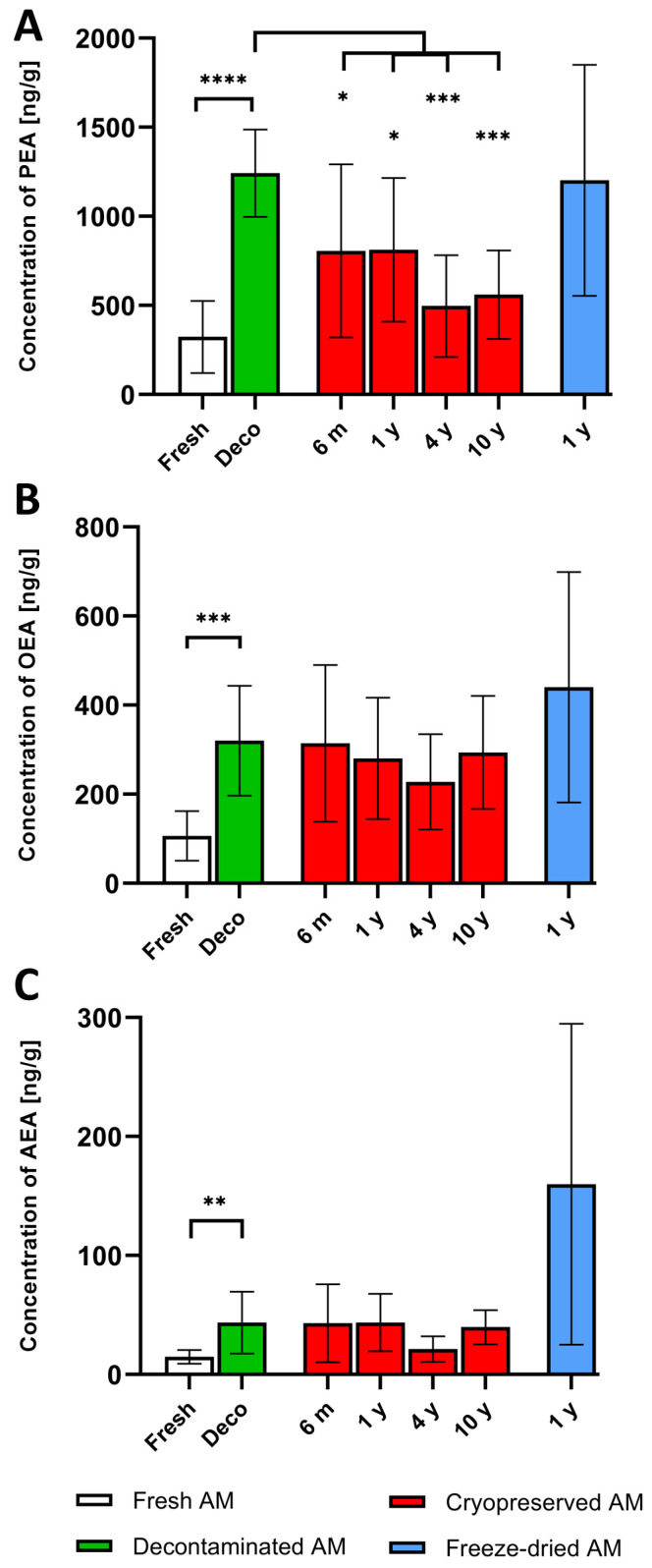
The levels of N-acylethanolamines in fresh, decontaminated, cryopreserved, and freeze-dried amniotic membranes. The average concentrations of palmitoylethanolamide (PEA) (**A**), oleoylethanolamide (OEA) (**B**), and anandamide (AEA) (**C**) are expressed as ng/g. *p*-value: *p* < 0.05 *; *p* < 0.01 **; *p* < 0.001 ***; *p* < 0.0001 ****, m = months, y = years.

NAEs were detected in all analyzed samples. The highest levels were measured for PEA. Values of individual NAEs in cryopreserved and freeze-dried samples stored for different time periods were compared with values measured after decontamination as all stored AM were decontaminated during tissue processing. A significant increase in all NAE values compared to fresh samples was detected after 24 h decontamination. These were used as baseline values for comparison with cryopreserved and freeze-dried samples. The average concentrations of PEA, OEA, and AEA are shown in Table 1 and Figure 2. The mean concentration of PEA in all cryopreserved specimens (six months, one, four, and 10 years) was significantly lower compared to decontaminated AM, Figure 2A. A different situation was observed for OEA and AEA, where no significant difference was found after cryopreservation, Figure 2B,C. However, significantly higher values for all NAEs were detected in cryopreserved samples compared to fresh tissue before decontamination, see Table S2, Figure S1 in Supplementary Materials. The concentration of particular NAEs in freeze-dried AM analyzed after one year were almost identical (PEA, AEA), or even higher (OEA) compared to baseline averages (decontaminated AM), but no significant difference was found, Figure 2.

### 3.2. The Detection of PPAR-α Receptor Using Indirect Immunohistochemistry

**Figure 3:**
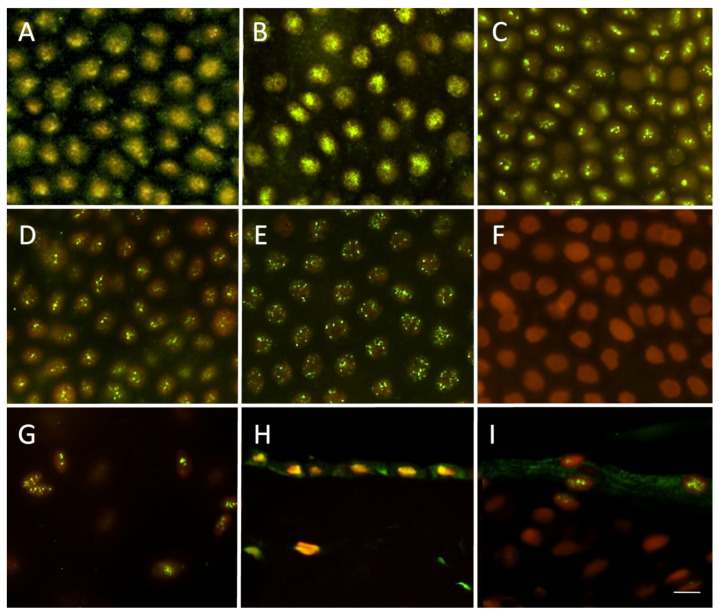
Expression of PPAR-α receptor in amniotic membrane. Immunostaining in fresh (**A**), decontaminated (**B**), cryopreserved stored for one year (**C**), cryopreserved stored for 4 years (**D**), freeze-dried for one-year stored amniotic membrane samples (**E**), negative control–amniotic membrane cryopreserved for one year (**F**). Strong nuclear staining for PPAR-α receptor (green) was detected in 95–100% of epithelial cells (**A**–**E**). The nuclear staining was also observed in the mesenchymal cells of underlying fibroblast layer (**G**). The presence of PPAR-α was confirmed on amniotic membrane cryosections (**H**). Human cryosections of arm skin were used as a positive control (**I**). Nuclei were counterstained with propidium-iodide (red). Scale bar represents 10 μm.

The PPAR-α receptor was found in all tested tissue. The strong immunostaining was homogenously present through 98–100% of nuclei in the epithelial layer of AM. The positivity was also observed in mesenchymal cells of the fibroblast layer. The nuclear positivity was more diffused in fresh and decontaminated tissue, while more prominent dot signals were present in cryopreserved and freeze-dried samples, see Figure 3.

## 4. Discussion

The main goal of this study was to determine whether the analgesic and anti-inflammatory endogenous lipid mediators NAEs present in AM allografts persist under standard tissue bank storage conditions. We have shown that there is significant decrease of PEA in cryopreserved AM stored from six months to ten years. No further significant decreases in either PEA, AEA, or OEA between baseline levels and cryopreserved or freeze-dried AM allografts stored from six months up to the longest tested period were detected.

The presence of NAEs in placental membranes was first described by Marczylo in 2009, who detected anandamide (AEA) in amniochorionic membrane and placenta [59]. In addition, pain relief has been repeatedly described both after the application of AM on the damaged or diseased ocular surface [60,61], and also on the surface of various types of wounds [33,62]. However, only recently has the presence of NAEs been linked to the analgesic effect of AM allografts [40]. In addition to the analgesic effect, NAEs can also participate in nociceptive, anti-inflammatory, and neuroprotective effects [41,45,49,50,51], which all of which are linked to AM properties receptor [42].

Previously, we have shown that the content of PEA, AEA, and OEA in placental samples is variable, mainly because of the known inter-individual differences between donors, but their concentrations can be increased by tissue decontamination [40]. This effect is probably caused by both the production of NAEs by surviving cells in AM and their release from degrading tissue [40]. Results from the first set of placentas (1–9) confirmed the increase of all NAEs after 24 h of decontamination with an antibiotic solution. For PEA, the average increase was 3.8-fold, for OEA 3-fold, and for AEA 2.9-fold. This indicates that all tested NAEs react to decontamination in a similar way. Given that the decontamination was an integral part of the preparation of all placentas that were used for the second part of the experiment (placentas 10–18), the mean concentration of particular NAEs in decontaminated samples then served as baselines.

Although cryopreservation resulted in a significant decrease of PEA after all storage periods, OEA and AEA were not significantly changed. On the other hand, the level of PEA in cryopreserved tissue after different storage time periods was significantly higher compared to the average measured in fresh, i.e., in non-decontaminated tissue. The only exception was following four years of incubation, where the value was higher, but with no statistical significance, see Supplementary data, Table S2. For all NAEs, there was no significant decrease between six months to ten years of storage, which indicates that PEA degradation is more dependent on the freezing or melting process rather than the length of cryopreservation. The decrease of PEA concentrations after cryopreservation can be explained by higher sensitivity of this enzyme compared to OEA or AEA. Another possibility is activation of enzymes degrading NAEs in AM [4,5].

Due to the six-month quarantine period, after which the allografts are released for transplantation, the most important NAE values are between the first and fourth year, i.e., in the period during which tissue can be used for grafting before expiration. The cryopreserved and freeze-dried AM allografts are typically stored for between two to five years prior to expiration [4,5].

Very interesting values of NAEs were found after twelve months of storage of freeze-dried AM. The measured concentrations were practically identical to decontaminated tissue (PEA), or even higher (OEA, AEA), although the differences were not significant. Given that no longer-preserved freeze-dried tissue was available under the conditions of our tissue bank, it is necessary to verify these data on a larger number of AM samples and also after a longer time period. 

We noticed a decrease in PEA concentration in cryopreserved samples, therefore, we wanted to find out if there are any data available on the analgesic effects of AM depending on its type. To the best of our knowledge, no study has compared the analgesic effect between multiple types of AM. Of the studies where pain was assessed using a pain score, analgesic effect was verified in studies using fresh [16,17], cryopreserved [33,62,63], and air-dried terminally sterilized (gamma irradiation 25 k Gy) [34] AM allografts for various wound treatment.

It has been shown, that NAEs exert their actions via several mechanisms [64], particularly via the PPAR-α receptor [42]. We found that the PPAR-α receptor remained preserved in all samples of AM allograft used, regardless of the type and length of storage. Regarding correlation between PPAR-α receptor and NAE synthesis, the expression of PPAR-α in AM only confirmed the synthesis of NAEs in this tissue, which is caused by their autacoid nature (locally produced and acting regulating molecules) [44]. Therefore, from the point of view of the analgesic effect, the expression of NAEs in the skin or in another tissue, for example on the surface of the eye, where AM is applied during treatment, is important. The expression of PPAR-α in the skin, (which is treated by AM), was recently demonstrated in normal and hypertrophic skin scars during the wound healing process [50]. In the presented study, the expression was confirmed in healthy arm skin (Figure 3I).

## 5. Conclusions

We analyzed the content of NAEs in long-term stored AM grafts prepared for grafting and have shown that in cryopreserved tissue, PEA, OEA, and AEA remain in relatively high concentrations even after four years of storage. This means that the above-described features of all three NAEs may be involved in positive analgesic and anti-inflammatory effects of AM allografts during wound healing. 

## Figures and Tables

**Table 1:** Concentrations of palmitoylethanolamide (PEA), oleoylethanolamide (OEA), and anandamide (AEA) (ng/g) in amniotic membranes in fresh, decontaminated (Deco), cryopreserved (Cryo), and freeze-dried (F-Dry) specimens. Cryopreserved samples were stored for six months (m) and for one-, four-, and ten-years (y). Freeze-dried samples were stored for one year.

Samples	Fresh	Deco	Cryo 6 m	Cryo 1 y	Cryo 4 y	Cryo 10 y	F-Dry 1 y
PEA (AV)	323.85	1241.76	806.26	812.86	497.16	561.16	1121.76
PEA (SD)	202.68	245.07	485.69	403.27	285.76	248.25	692.50
OEA (AV)	106.56	320.42	314.30	280.56	227.87	293.77	440.25
OEA (SD)	55.68	123.26	175.76	136.40	106.84	127.06	258.60
AEA (AV)	14.87	43.58	43.19	43.70	21.31	39.76	160.00
AEA (SD)	5.82	26.02	32.75	24.09	10.74	14.39	134.99

Data are presented as an average value (AV). SD, standard deviation.

## Data Availability

Data is contained within the article or supplementary material.

## Supplementary Materials

The following supporting information can be downloaded at: https://www.mdpi.com/article/10.3390/bioengineering10060740/s1. Supplementary data, Table S1: Concentrations of palmitoylethanolamide (PEA), oleoylethanolamide (OEA), and anandamide (AEA) (ng/g) in amniotic membranes in fresh, decontaminated (Deco), cryopreserved (Cryo), and freeze-dried (F-Dry) specimens. Statistical significance between decontaminated and other samples: fresh, cryopreserved, freeze-dried. Footer: Cryopreserved samples were stored for six months (m) and for one-, four-, and ten-years (y). Freeze-dried samples were stored for one year. Data are presented as an average value (AV). SD, standard deviation. Table S2. Concentrations of palmitoylethanolamide (PEA), oleoylethanolamide (OEA), and anandamide (AEA) (ng/g) in amniotic membranes in fresh, decontaminated (Deco), cryopreserved (Cryo), and freeze-dried (F-Dry) specimens. Statistical significance between fresh and other samples (cryopreserved, freeze-dried). Footer: Cryopreserved samples were stored for six months (m) and for one-, four-, and ten-years (y). Freeze-dried samples were stored for one year. Data are presented as an average value (AV). SD, standard deviation. Figure S1: The levels of N-acylethanolamines in fresh, cryopreserved and freeze-dried amniotic membranes. The average concentrations of palmitoylethanolamide (PEA) (A), oleoylethanolamide (OEA) (B), and anandamide (AEA) (C) are expressed in ng/g. *p*-value: *p* < 0.05 *; *p* < 0.01 **; *p* < 0.001 ***; *p* < 0.0001 ****, m = months, y = years. Statistical significance between fresh and other samples (cryopreserved, freeze-dried).
