# Supplementary material for: Quantification of Analgesic and Anti-Inflammatory Lipid Mediators in Long-Term Cryopreserved and Freeze-Dried Preserved Human Amniotic Membrane
Source: Bioengineering (Basel). 2023 Jun 20;10(6):740. doi: 10.3390/bioengineering10060740 (PMC10294941; doi:10.3390/bioengineering10060740)
Supplement: Supplementary file 1 [file bioengineering-10-00740-s001.zip › bioengineering-2437729-supplementary.pdf]

bioengineering

MDPI

Supplementary Materials

Quantification of Analgesic and Anti-Inflammatory Lipid Mediators in Long-Term Cryopreserved and Freeze-Dried Preserved Human Amniotic Membrane

Vladimir Vrkoslav [1,†], Ingrida Smeringaiova [2,†], Natalia Smorodinova [2,3,†], Alzbeta Svobodova [4], Stepan Strnad [1], Catherine Joan Jackson [5], Jan Burkert [6] and Katerina Jirsova [2,†,*]

[1] The Institute of Organic Chemistry and Biochemistry of the Czech Academy of Sciences, 160 00 Prague, Czech Republic; vrkoslav@uochb.cas.cz (V.V.); stepan.strnad@uochb.cas.cz (S.S.)
[2] Laboratory of the Biology and Pathology of the Eye, Institute of Biology and Medical Genetics, First Faculty of Medicine, Charles University and General University Hospital in Prague, 128 01 Prague, Czech Republic
[3] Institute of Histology and Embryology, First Faculty of Medicine, Charles University and General University Hospital in Prague, 128 01 Prague, Czech Republic
[4] 2nd Department of Surgery—Department of Cardiovascular Surgery, First Faculty of Medicine, Charles University and General University Hospital in Prague, 128 08, Prague, Czech Republic; alzbeta.svo@seznam.cz
[5] Department of Medical Biochemistry, Oslo University Hospital and Institute of Oral Biology, University of Oslo, 0316 Oslo, Norway
[6] Department of Transplantation and Tissue Bank, University Hospital in Motol, 150 06 Prague, Czech Republic
* Correspondence: katerina.jirsova@lf1.cuni.cz; Tel.: +420-224-968-006
† These authors contributed equally to this work.

Table S1. Concentrations of palmitoylethanolamide (PEA), oleoylethanolamide (OEA), and anandamide (AEA) (ng/g) in amniotic membranes in fresh, decontaminated (Deco), cryopreserved (Cryo), and freeze-dried (F-Dry) specimens. Statistical significance between decontaminated and other samples: fresh, cryopreserved, freeze-dried.

PEA							
**Placenta**	**Fresh**	**Deco**	**Cryo 6 m**	**Cryo 1 y**	**Cryo 4 y**	**Cryo 10 y**	**F-Dry 1 y**
**P1**	217.55	1487.36	1817.50	1362.33	991.33	267.00	2013.88
**P2**	239.34	1383.82	684.00	1273.33	569.83	351.00	906.99
**P3**	148.16	1218.37	895.67	497.07	243.12	667.74	603.46
**P4**	662.22	1369.13	627.75	247.97	261.30	433.21	1094.44
**P5**	395.33	1502.71	321.27	387.19	280.48	748.00	500.76
**P6**	171.08	1169.34	485.80	590.37	881.50	900.00	2195.56
**P7**	258.84	768.35	811.82	992.50	261.90	n/a	1347.16
**P8**	652.88	958.97	n/a	828.00	622.00	n/a	311.84
**P9**	169.26	1317.81	n/a	1137.00	363.00	n/a	n/a
**AV**	323.85	1241.76	806.26	812.86	497.16	561.16	1121.76
**SD**	202.68	245.07	485.69	403.27	285.76	248.25	692.50
**Mann-Whitney U test**							
**Samples**		***P* value**	**Significance**				
**Deco vs Fresh**		<0.0001	****				
**Deco vs Cryo 6 m**		0.0311	*				
**Deco vs Cryo 1 y**		0.0180	*				
**Deco vs Cryo 4 y**		0.0003	***				
**Deco vs Cryo 10 y**		0.0008	***				
**Deco vs F-Dry 1 y**		0.5071	ns				
**OEA**							

Placenta	Fresh	Deco	Cryo 6 m	Cryo 1 y	Cryo 4 y	Cryo 10 y	F-Dry 1 y
P1	126.75	174.31	593.80	417.34	474.31	326.07	798.47
P2	115.26	324.58	211.28	462.89	266.73	236.90	239.01
P3	59.80	241.46	367.03	141.24	224.37	338.00	153.30
P4	150.75	294.67	489.60	134.50	223.04	454.44	349.36
P5	101.69	404.21	110.89	206.90	261.90	113.42	365.83
P6	65.57	591.05	186.17	210.90	187.25	n/a	n/a
P7	67.96	218.22	241.35	158.12	113.42	n/a	803.17
P8	223.06	353.50	n/a	352.09	171.88	n/a	372.64
P9	48.17	281.82	n/a	441.05	127.88	n/a	n/a
AV	106.56	320.42	314.30	280.56	227.87	293.77	440.25
SD	55.68	123.26	175.76	136.40	106.84	127.06	258.60
**Mann-Whitney U test**							
**Samples**		***P* value**	**Significance**				
**Deco vs Fresh**		0.0002	***				
**Deco vs Cryo 6 m**		0.7577	ns				
**Deco vs Cryo 1 y**		0.4894	ns				
**Deco vs Cryo 4 y**		0.0625	ns				
**Deco vs Cryo 10 y**		>0.9999	ns				
**Deco vs F-Dry 1 y**		0.4079	ns				
**AEA**							
**Placenta**	**Fresh**	**Deco**	**Cryo 6 m**	**Cryo 1 y**	**Cryo 4 y**	**Cryo 10 y**	**F-Dry 1 y**
P1	11.67	20.43	109.17	58.46	33.14	30.28	317.23
P2	12.71	36.45	26.10	61.87	26.19	29.29	36.67
P3	4.98	11.74	59.98	24.84	13.94	39.19	24.23
P4	17.77	22.60	16.02	15.24	13.43	60.30	60.98
P5	9.28	37.47	19.04	27.21	35.08	n/a	223.28
P6 (P11)	17.92	87.07	29.61	31.03	7.52	n/a	n/a
P7	17.71	75.42	42.43	27.33	n/a	n/a	n/a
P8 (P10)	17.09	37.52	n/a	59.00	12.03	n/a	297.60
P9	24.69	63.51	n/a	88.35	29.12	n/a	n/a
AV	14.87	43.58	43.19	43.70	21.31	39.76	160.00
SD	5.82	26.02	32.75	24.09	10.74	14.39	134.99
**Mann-Whitney U test**							
**Samples**		***P* value**	**Significance**				
**Deco vs Fresh**		0.0028	**				
**Deco vs Cryo 6 m**		0.8371	ns				
**Deco vs Cryo 1 y**		>0.9999	ns				
**Deco vs Cryo 4 y**		0.0464	ns				
**Deco vs Cryo 10 y**		>0.9999	ns				
**Deco vs F-Dry 1 y**		0.1447	ns				

Cryopreserved samples were stored for six months (m) and for one-, four-, and ten-years (y). Freeze-dried samples were stored for one year. Data are presented as an average value (AV). SD, standard deviation.

Table S2. Concentrations of palmitoylethanolamide (PEA), oleoylethanolamide (OEA), and anandamide (AEA) (ng/g) in amniotic membranes in fresh, decontaminated (Deco), cryopreserved (Cryo), and freeze-dried (F-Dry) specimens. Statistical significance between fresh and other samples (cryopreserved, freeze-dried).

**PEA**							
**Placenta**	**Fresh**	**Deco**	**Cryo 6 m**	**Cryo 1 y**	**Cryo 4 y**	**Cryo 10 y**	**F-Dry 1 y**
**P1**	217.55	1487.36	1817.50	1362.33	991.33	267.00	2013.88
**P2**	239.34	1383.82	684.00	1273.33	569.83	351.00	906.99
**P3**	148.16	1218.37	895.67	497.07	243.12	667.74	603.46
**P4**	662.22	1369.13	627.75	247.97	261.30	433.21	1094.44
**P5**	395.33	1502.71	321.27	387.19	280.48	748.00	500.76
**P6**	171.08	1169.34	485.80	590.37	881.50	900.00	2195.56
**P7**	258.84	768.35	811.82	992.50	261.90	n/a	1347.16
**P8**	652.88	958.97	n/a	828.00	622.00	n/a	311.84
**P9**	169.26	1317.81	n/a	1137.00	363.00	n/a	n/a
**AV**	323.85	1241.76	806.26	812.86	497.16	561.16	1121.76
**SD**	202.68	245.07	485.69	403.27	285.76	248.25	692.50
**Mann-Whitney U test**							
**Samples**		***P* value**	**Significance**				
**Deco vs Fresh**		<0.0001	****				
**Fresh vs Cryo 6 m**		0.0079	**				
**Fresh vs Cryo 1 y**		0.0078	**				
**Fresh vs Cryo 4 y**		0.0770	ns				
**Freshvs Cryo 10 y**		0.0256	*				
**Fresh vs F-Dry 1 y**		0.0042	**				
**OEA**							
**Placenta**	**Fresh**	**Deco**	**Cryo 6 m**	**Cryo 1 y**	**Cryo 4 y**	**Cryo 10 y**	**F-Dry 1 y**
**P1**	126.75	174.31	593.80	417.34	474.31	326.07	798.47
**P2**	115.26	324.58	211.28	462.89	266.73	236.90	239.01
**P3**	59.80	241.46	367.03	141.24	224.37	338.00	153.30
**P4**	150.75	294.67	489.60	134.50	223.04	454.44	349.36
**P5**	101.69	404.21	110.89	206.90	261.90	113.42	365.83
**P6**	65.57	591.05	186.17	210.90	187.25	n/a	n/a
**P7**	67.96	218.22	241.35	158.12	113.42	n/a	803.17
**P8**	223.06	353.50	n/a	352.09	171.88	n/a	372.64
**P9**	48.17	281.82	n/a	441.05	127.88	n/a	n/a
**AV**	106.56	320.42	314.30	280.56	227.87	293.77	440.25
**SD**	55.68	123.26	175.76	136.40	106.84	127.06	258.60
**Mann-Whitney U test**							
**Samples**		***P* value**	**Significance**				
**Deco vs Fresh**		0.0002	***				
**Fresh vs Cryo 6 m**		0.0052	**				
**Fresh vs Cryo 1 y**		0.0019	**				
**Fresh vs Cryo 4 y**		0.0040	**				
**Fresh vs Cryo 10 y**		0.0120	*				
**Fresh vs F-Dry 1 y**		0.0003	***				
**AEA**							
**Placenta**	**Fresh**	**Deco**	**Cryo 6 m**	**Cryo 1 y**	**Cryo 4 y**	**Cryo 10 y**	**F-Dry 1 y**
**P1**	11.67	20.43	109.17	58.46	33.14	30.28	317.23
**P2**	12.71	36.45	26.10	61.87	26.19	29.29	36.67

**P3**	4.98	11.74	59.98	24.84	13.94	39.19	24.23
**P4**	17.77	22.60	16.02	15.24	13.43	60.30	60.98
**P5**	9.28	37.47	19.04	27.21	35.08	n/a	223.28
**P6 (P11)**	17.92	87.07	29.61	31.03	7.52	n/a	n/a
**P7**	17.71	75.42	42.43	27.33	n/a	n/a	n/a
**P8 (P10)**	17.09	37.52	n/a	59.00	12.03	n/a	297.60
**P9**	24.69	63.51	n/a	88.35	29.12	n/a	n/a
**AV**	14.87	43.58	43.19	43.70	21.31	39.76	160.00
**SD**	5.82	26.02	32.75	24.09	10.74	14.39	134.99
**Mann-Whitney U test**							
**Samples**		***P* value**	**Significance**				
**Deco vs Fresh**		0.0028	**				
**Fresh vs Cryo 6 m**		0.0052	**				
**Fresh vs Cryo 1 y**		0.0008	***				
**Fresh vs Cryo 4 y**		0.2766	ns				
**Fresh vs Cryo 10 y**		0.0028	**				
**Fresh vs F-Dry 1 y**		0.0008	***				

Footer: Cryopreserved samples were stored for six months (m) and for one-, four-, and ten-years (y). Freeze-dried samples were stored for one year. Data are presented as an average value (AV). SD, standard deviation.

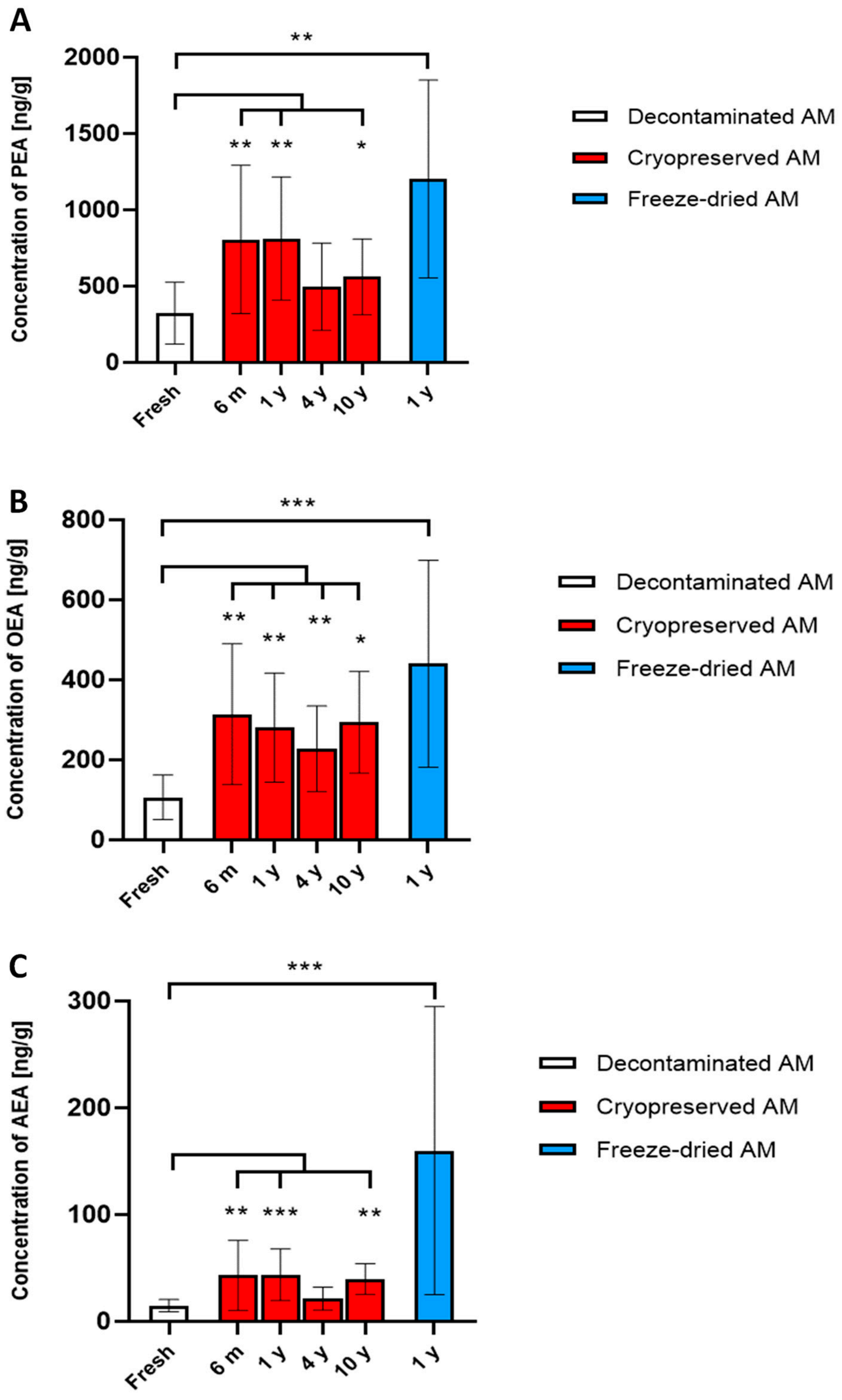

Figure S1. The levels of N-acylethanolamines in fresh, cryopreserved and freeze-dried amniotic membranes. The average concentrations of palmitoylethanolamide (PEA) (**A**), oleoylethanolamide (OEA) (**B**), and anandamide (AEA) (**C**) are expressed in ng/g. P-value: P < 0.05*; P < 0.01**; P < 0.001***; P < 0.0001****, m = months, y = years. Statistical significance between fresh and other samples (cryopreserved, freeze-dried). Cryopreserved samples were stored for six months (m) and for one-, four-, and ten-years (y). Freeze-dried samples were stored for one year. Data are presented as an average value (AV). SD, standard deviation.
